# Health Tract, No. 93

**Published:** 1865

**Authors:** 


### HEALTH TRACT, No. 93.

### CHILD BEARING.

**WHEN BEGAN WE?**—We end never! for the soul is immortal, and can not die. When the soul's existence commences is as yet a conjecture. Nor can we tell when the immaterial first takes up its dwelling with the material; when the soul enters the body. But this we do know, that, at a point when the man that is to be is so minute as to require the microscope to determine whether it exists or not, the first faint outlines of the new being are defined to be a nervous system. The very first step cognizable to us, which nature takes to make a man a living soul, is to prepare the machinery, so to speak, through which that soul is to manifest itself. It is the nervous system which first begins to live, and to appropriate to itself those materials of growth which eventually become the human body and make a man. Nothing can be clearer than that the nervous system of the new being is connected with and is dependent on that of the parent, and that the hues, the impressions of the young, depend on the character of those of the parent. If, at this time, the parent is in perfect health, and so remains, it is fair to presume that the child will be born in perfect health, body and mind. These statements make the strongest possible appeal to all who may become mothers, to make it their constant study, their steady aim and effort, to secure a healthful condition of the body and a state of mind which shall be uniformly all that the mother desires the child to possess—piety, integrity, dignity, and an elevation of soul, which proves relationship to the Infinite. If the mother that is to be, wishes her child to possess vigorous manly health, she must cultivate the strictest personal cleanliness, extending to the most minute item pertaining to the human body; she must eat with regularity, not oftener than thrice a day; she must keep her feet, by all possible means, always dry and warm; her sleep must be early, and of the greatest abundance that nature can possibly take, out of daylight; one half of each day should be spent in open air activities; and nearly all the time of in-doors should be employed in cheerful, interesting, active work, constantly diversified, so as not to overtax one set of muscles, and leave others comparatively idle. The very best course to pursue is, to take a part in every thing going on, in fact, "every thing by turns, but nothing long." One of the most important items of advice that can be given in this connection is, that an hour or two should be spent in walking in the open air, at two or three different times, until the very last. Nothing so certainly and so safely, contributes to an easy deliverance. And these, with regular, daily, bodily habits, would add incalculably to the sum total of human happiness; whilst by their neglect, by simply passing the time in eating, lounging, and listlessness, in the wearing, irritating inactivities of a boarding-house, or hotel life, monsters in bodily shape, and imbeciles in mind, are constantly thrown out on society, to be disgusted by their presence, or to be taxed by their confinement in some insane asylum.

